# Phylogenetic surveillance of travel-related Zika virus infections through whole-genome sequencing methods

**DOI:** 10.1038/s41598-019-52613-8

**Published:** 2019-11-11

**Authors:** Kimia Kamelian, Vincent Montoya, Andrea Olmstead, Winnie Dong, Richard Harrigan, Muhammad Morshed, Jeffrey B. Joy

**Affiliations:** 1 University of British Columbia, Division of AIDS, Department of Medicine, Vancouver, BC Canada; 2 BC Centre for Excellence in HIV/AIDS, Vancouver, BC Canada; 3 BC Centre for Disease Control Public Health Laboratory, Vancouver, BC Canada; 4 University of British Columbia, Department of Pathology and Laboratory Medicine, Vancouver, BC Canada; 5 University of British Columbia, Division of Infectious Diseases, Department of Medicine, Vancouver, BC Canada

**Keywords:** Phylogenetics, Viral infection

## Abstract

In 2018, the World Health Organization identified the Zika virus (ZIKV) as a pathogen that should be prioritized for public health research due to its epidemic potential. In this study, whole-genome sequencing (WGS) of travel-acquired ZIKV infections was used to examine the limitations of phylogenetic analysis. WGS and phylogenetic analysis were performed to investigate geographic clustering of samples from five Canadians with travel-acquired ZIKV infections and to assess the limitations of phylogenetic analysis of ZIKV sequences using a phylogenetic cluster approach. Genomic variability of ZIKV samples was assessed and for context, compared with hepatitis C virus (HCV) samples. Phylogenetic analysis confirmed the suspected region of ZIKV infection for one of five samples and one sample failed to cluster with sequences from its suspected country of infection. Travel-acquired ZIKV samples depicted low genomic variability relative to HCV samples. A floating patristic distance threshold classified all pre-2000 ZIKV sequences into separate clusters, while only Cambodian, Peruvian, Malaysian, and South Korean sequences were similarly classifiable. While phylogenetic analysis of ZIKV data can identify the broad geographical region of ZIKV infection, ZIKV’s low genomic variability is likely to limit precise interpretations of phylogenetic analysis of the origins of travel-related cases.

## Introduction

In 2018, the World Health Organization (WHO) held the second annual review of R&D Blueprint, identifying the Zika virus (ZIKV) as a pathogen that should be prioritized for research and development in a public health emergency context, due to its epidemic potential and its lack of sufficient treatment^1^. First identified in Uganda in 1947, ZIKV is a member of the *Flavivirus* genus within the *Flaviviridae* family and is an arthropod-borne virus spread primarily through infected *Aedes* mosquitoes^2^. Sequencing analyses involving whole-genome sequences and gene-specific analyses have identified three lineages: Asian, East African, and West African^2^. Phylogenetic and molecular clock analyses have confirmed the Asian lineage is responsible for the recent sporadic spread of ZIKV outside of Africa and Asia: Yap Island, Federated States of Micronesia in 2007; French Polynesia in 2013; and Brazil in 2015^3–5^. The emergence of ZIKV in recent years has been associated with increased incidence of the neurological conditions Guillain-Barré syndrome and meningoencephalitis, and prenatal microcephaly^6–9^. Similar to infections by other members of the *Flavivirus* genus as well as chikungunya virus, individuals infected with ZIKV are generally asymptomatic, with only one in five infected individuals showing non-specific symptoms such as a mild fever, rash, and conjunctivitis^6,10^.

The majority of ZIKV infections in regions without significant prevalence of ZIKV vector *Aedes* mosquitos, such as Canada, are travel-acquired infections^11^. Routine surveillance to identify and track new cases of ZIKV infections currently rely on suspicions of ZIKV infection by healthcare providers, and additional laboratory confirmation through real time reverse transcription-polymerase chain reaction (RT-PCR) amplification and antibody-based tests^6,12^. However, ZIKV may be misdiagnosed as other, closely related infections due to the non-specific nature of ZIKV symptoms. Moreover, laboratory confirmation through real time RT-PCR can be limited in low-resource settings while serological antibody-based tests may be cross-reactive among *flaviviruses* leading to further misdiagnosis of ZIKV infections^12^.

Whole-genome sequencing (WGS) methods have previously been used to analyze the evolution and genomic variability of viruses such as hepatitis C virus (HCV)^13^, and are progressively being used during viral outbreaks in an effort to identify transmission patterns^14–17^. Sequencing of ZIKV presents an alternative or Supplementary Method of ZIKV surveillance and may allow insight into the geographic origins of infections, transmission patterns, and genomic diversification. In this study, through phylogenetic analysis of whole-genome sequences derived from patients with confirmed travel-acquired ZIKV infection, we attempt to identify the origins of travel-acquired ZIKV infections and, through intraspecies and intrafamilial comparative analysis with HCV, examine ZIKV’s genomic variability.

## Results

### Whole-genome sequencing results from travel-acquired ZIKV infections

Samples with higher ZIKV Ct values had a higher number of human reads and lower percentage of ZIKV reads (Pearson’s correlation: −0.99, p < 0.05), consistent with a lower absolute amount of ZIKV in the samples. Higher Ct values were also associated with lower overall depth of ZIKV coverage (Pearson’s correlation: −0.88, p < 0.05) (Table 1). Each sample’s consensus sequence ranged in length from 8–10.5 kilo-base-pair (kbp). Median depth of coverage of all samples was 24,000 reads (interquartile range [IQR]: 17,000–25,000). Although some sequences had low depth of coverage (fewer than 10 reads), they still provided sufficient genome coverage for the regions sequenced. The GenBank accession numbers for the five Canadian samples obtained from individuals with travel-related ZIKV infections (putatively from Belize, Mexico, an undisclosed Caribbean region, Barbados, and Panama) are as follows: Sample 1 – Belize, MN473450; Sample 2 – Mexico, MN473451; Sample 3 – Caribbean, MN473452; Sample 4 – Barbados, MN473453; and Sample 5 – Panama, MN473454.

**Table 1:** Sequencing results of five samples with confirmed travel-acquired ZIKV infection.

Sample^a^	Location of Infection	Ct Value^b^	No. of Reads^c^	No. of Human Reads	No. of ZIKV Reads	% ZIKV Virus	Median Depth of Coverage^d^
Sample 1	Belize	33	3,342,514	737,467	1,442,159	43	673
Sample 2	Mexico	28	3,052,866	7,863	2,532,761	83	25143
Sample 3	Caribbean	30	3,088,142	73,386	2,427,755	79	23884
Sample 4	Barbados	26	2,046,216	1,774	1,672,980	82	16832
Sample 5	Panama	21	3,282,070	4,388	2,629,877	80	32939

^a^Samples were provided by the BCCDC and were anonymized again at the BC-CfE.

^b^Cyclic threshold (Ct) was obtained through quantitative RT-PCR.

^c^Total number of human, virus, and random reads obtained per sample.

^d^Median depth of coverage was calculated using the median number of reads across the genome for each sample.

### Phylogenetic analysis of travel-acquired ZIKV infections

**Figure 1:**
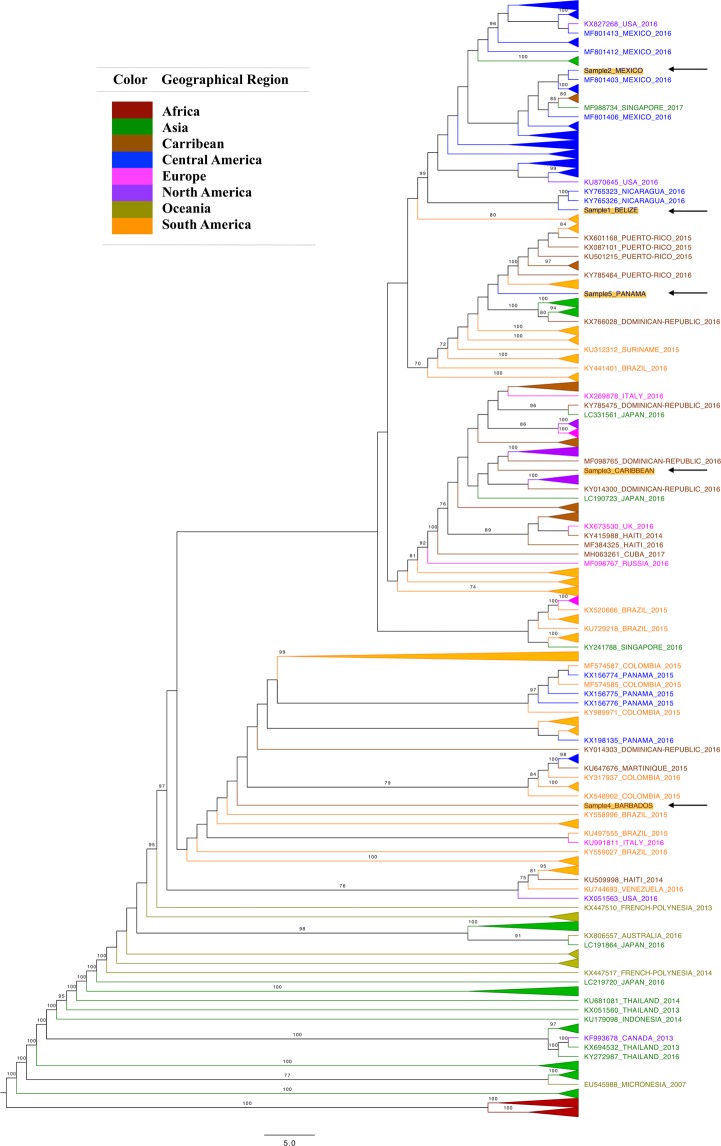
Phylogenetic analysis of five samples with confirmed ZIKV infection collected by the BCCDC. Phylogenetic analysis of Canadian travel-related clinical cases of ZIKV infection with geographically annotated reference sequences from GenBank confirmed the suspected region of ZIKV infection for one (Sample 2 – Mexico) of the five samples. Two samples, Sample 1 – Belize and Sample 4 – Barbados, were missing whole-genome GenBank sequences from suspected areas of infection. However, they clustered within close geographical proximity to neighboring regions. The majority of GenBank reference sequences used in this study were from 2016 (n = 212) followed by 2015 (n = 77). Values for bootstraps greater than 70 are shown at the nodes. Scalebar shows the genetic difference through number of substitutions per site. Branches corresponding to taxa from the same geographical region were collapsed. An imbalance of taxa among different clades is observed within the tree topology.

Phylogenetic analysis of the resulting consensus sequences with whole-genome reference sequences obtained from GenBank confirmed the clinically indicated region of ZIKV infection for one (Sample 2 – Mexico) of the five samples (Fig. 1). Sample 1 – Belize and Sample 4 – Barbados were missing whole-genome reference sequences from clinically indicated areas of infection. However, they clustered within close geographical proximity to neighboring regions (Sample 1 had suspected travel-acquired ZIKV from Belize and clustered closely to samples from neighboring country Nicaragua, and Sample 4 had suspected travel-acquired ZIKV from Barbados and clustered closely to samples from Colombia) (Fig. 1). Although Sample 3 – Caribbean clustered closely with samples from the United States of America (USA) and samples from the Dominican Republic, it did not have a suspected country of infection and therefore phylogenetic analysis could not confirm or refute the suspected country of infection. Sample 5 – Panama failed to cluster to reference sequences from Panama, its suspected area of infection despite having full genome coverage. Relatively short branch lengths^18^ of the GenBank sequences and the Canadian travel-acquired ZIKV sequences implied limited genetic divergence between sequences from recent ZIKV cases.

### Genomic diversity of travel-acquired ZIKV infections

#### Nucleotide variation

**Figure 2:**
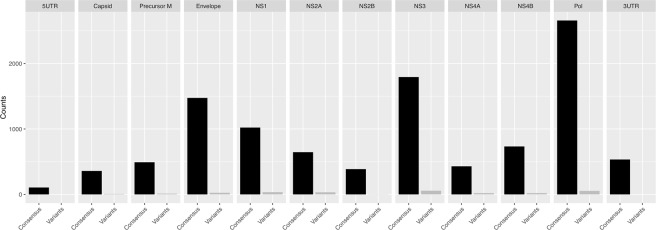
Nucleotide variation of samples. Nucleotide variation was measured for each sample as the number of consensus nucleotide to minor variants. Shown in Fig. 2, is the nucleotide variation averaged per gene over the samples sequenced in the study. In general, an overall relatively low nucleotide variation was observed despite the samples allegedly having five different origins of infections.

Nucleotide variation was measured for each Canadian sample as the average count and proportion of minority variants relative to the WHO ZIKV reference sequence from French Polynesia (GenBank accession number: KX369547)^19^. Shown in Fig. 2, is the nucleotide variation averaged per gene within the ZIKV genome over the five Canadian samples sequenced. Although minority variants were present throughout the ZIKV genome, of the 10,108 positions with at least 100 base-pair (bp) coverage, only 504 (4.9%) displayed any evidence of variation (Supplementary Fig. S1). In general, an overall relatively low nucleotide variation was observed in the patient sample set despite the samples having five different origins of ZIKV infection.

#### Shannon diversity index

**Figure 3:**
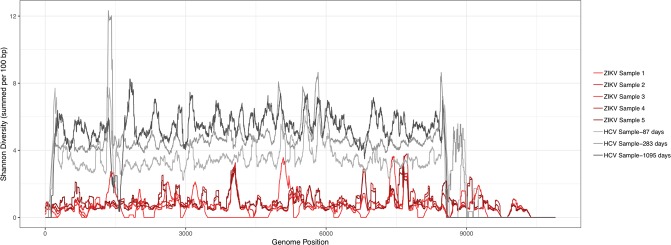
Shannon diversity index of ZIKV and HCV samples. The Shannon diversity index for ZIKV samples (n = 5) and HCV samples (n = 3) shows a relatively low diversity index of ZIKV samples compared to HCV samples. A sliding window approach was used to highlight diversification across all genomic motifs where diversities at each position were summed over each 100 base-pair window.

Diversity across the ZIKV genome was also calculated using the Shannon diversity index for all five of the Canadian travel-acquired samples (Fig. 3). The five ZIKV samples showed a relatively low diversity index when compared to the HCV samples.

### Country-specific genomic diversity of whole-genome ZIKV sequences

#### Patristic distance

Due to the challenges for geographic placement of the limited dataset of five Canadian ZIKV sample sequences, the remaining reference sequences selected in this study were also examined to determine the limitations of the phylogenetic signal in general for ZIKV consensus sequences. Here, the accuracy of the identification of each respective country for a given reference sequence was examined using a country-specific patristic distance threshold. For each country, specific thresholds were generated where the largest within-country phylogenetic distance (patristic distance) was compared with all between-country phylogenetic distances. When applying these country specific thresholds, only seven countries (Cambodia, Senegal, Peru, Uganda, South Korea, Nigeria, and Malaysia) were shown to have within-country patristic distances that were smaller than those distances between all other countries. Of these, four (Malaysia, South Korea, Peru, and Cambodia) belonged to the Asian lineage responsible for the recent epidemic. When examining the countries of the closest relatives for each of the 367 sequences in the phylogenetic tree, only 252 (68.7%) were isolated from the same country, indicating that the closest relative of 115 (31.3%) country-specific sequences were sequences isolated from another country.

#### Genomic variation

**Figure 4:**
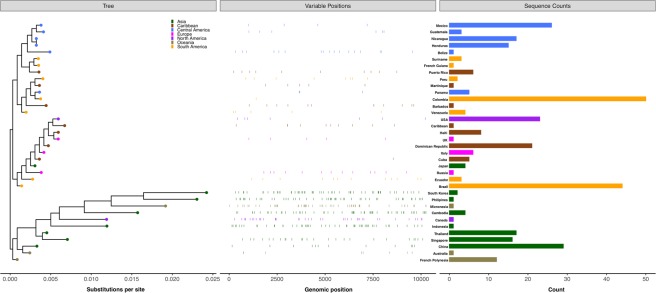
Nucleotide variability of ZIKV sequences after the year 2000 in dataset. Analysis of the genomic diversity of circulating ZIKV strains after the year 2000 revealed reduced genetic variability as the geographical expansion of ZIKV occurred from Asia to the Americas. A consensus sequence of the protein-coding regions was obtained for each country that had >1 whole-genome sequence (n = 24) and countries with a single representative sequence were also included in the analysis (n = 12). Countries with reference sequences acquired prior to the year 2000 were excluded (n = 5). Whole-genome sequences were aligned and rooted to the WHO reference from French Polynesia (GenBank accession number: KX369547). Variable positions were identified if they were present in >50% of all of the sequences corresponding to a specific country, but not present in the consensus sequence corresponding to any alternative country.

**Figure 5:**
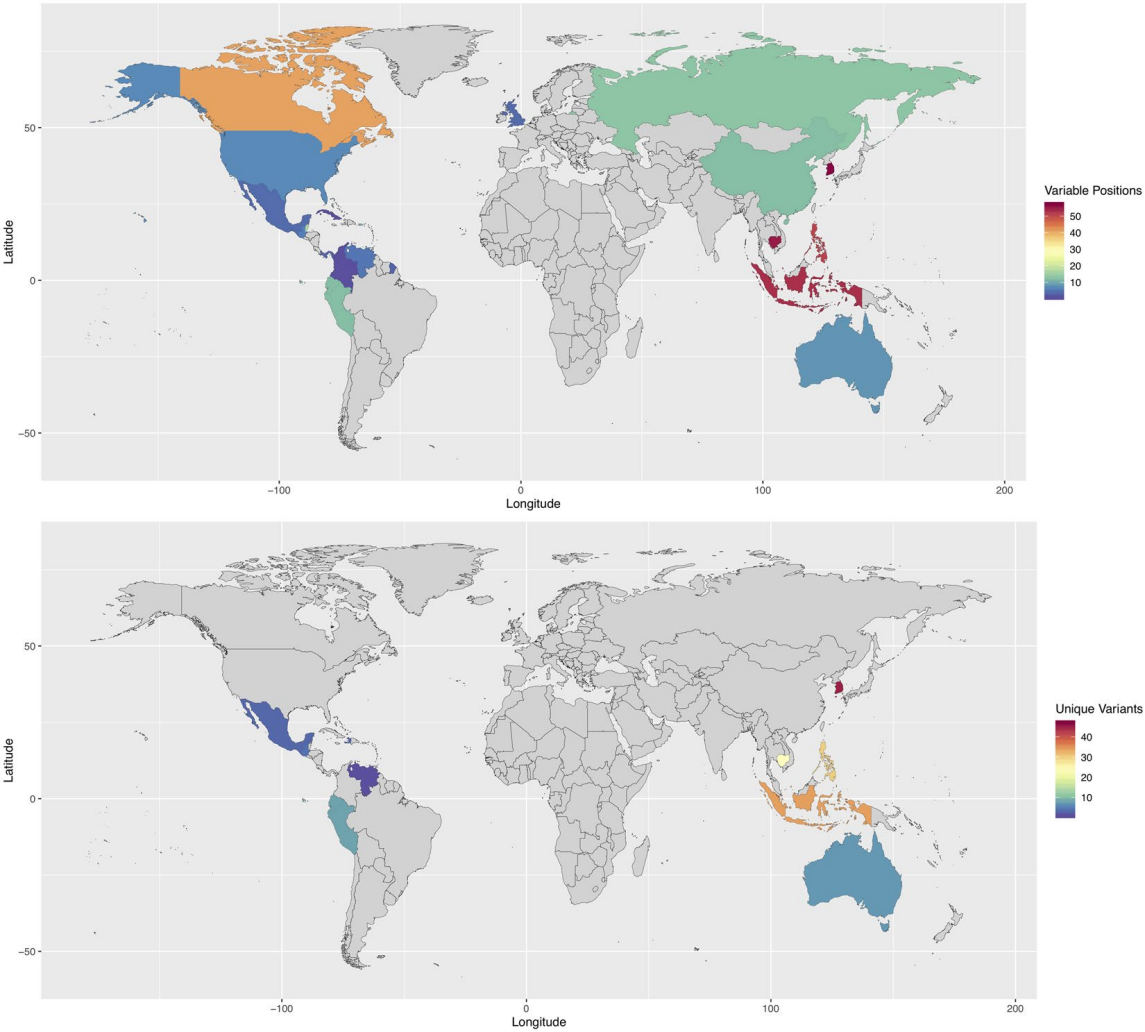
Country-specific nucleotide variants. (**A**) The quantity of variable positions for each country in the dataset are shown if they were present in >50% of all sequences corresponding to a specific country, but not present in the consensus sequence corresponding to any alternative country. (**B**) The number of nucleotide positions unique to a country is shown. Unique variants were identified relative to any individual sequences from alternative countries by comparing each variant to all nucleotides at the respective position. With geographical expansion into the Western Hemisphere and north- and southward spread of ZIKV, the number of unique variants identified in whole-genome sequences decreased.

To better understand the extent of ZIKV’s genetic variability as ZIKV infections spread between continents in the 21^st^ century, nucleotide diversity of whole-genome sequences isolated after the year 2000 were quantified, aligned and rooted to the WHO reference from French Polynesia (GenBank accession number: KX369547)^19^ (Fig. 4). Variable positions were identified if they were present in >50% of the sequences corresponding to a specific country, but not present in the consensus sequence of any alternative country. The number of variable positions decreased as ZIKV dispersed from Asia to the Americas (Fig. 5A). Variants unique to each country were further identified if they were present in sequences corresponding to a specific country, but not present in any sequences related to any other country (Fig. 5B). With geographical expansion and north- and southward spread of ZIKV, the number of nucleotides unique to each country decreased. In Asia, the highest number of unique variants were found in Micronesia (n = 47) followed by South Korea (n = 45) and the lowest were found in Singapore (n = 1). In the Americas, the highest number of unique variants were found in Ecuador and Peru (n = 8), and the lowest were found in Venezuela (n = 1) (Fig. 5B).

## Discussion

Surveillance of ZIKV is essential to the development of public health guidelines to prevent recurring and emerging ZIKV outbreaks, which are associated with growing numbers of neurological abnormalities and birth malformations^20,21^. Currently, ZIKV is detected through serological testing and real time RT-PCR, and these methods may not always be reliable^22,23^. In this proof-of-concept study, we performed WGS on five Canadian samples from the BCCDC with confirmed travel-acquired ZIKV infections, and aimed to verify the original countries of infections. Our findings suggest that although phylogenetic analysis of WGS data can generally identify the broad geographical region of ZIKV infection, it may not necessarily be used to pinpoint the exact country of infection due to the current circulating ZIKV and its low genomic variability, incomplete and biased coverage of reference sequences in databanks, and complications arising from extensive global travels.

While phylogenetic analysis did correctly verify the country associated with ZIKV infection for one (Sample 2 – Mexico) of the five samples, two samples (Sample 1 – Belize and Sample 4 – Barbados) were missing whole-genome reference sequences from suspected regions of ZIKV infections. Lack of WGS data from suspected regions of ZIKV infections on public, global databases does not necessarily indicate absence of ZIKV transmission in these populations. Limited resources in countries with suspected ZIKV infection^24^ and underreporting of ZIKV infections due to asymptomatic patients^25^ may contribute to the lack of data available on online resources even in the presence of local ZIKV transmission. Furthermore, inconsistent annotations of WGS data on public sequence databases such as GenBank, and the lack of curated and standardized available reference sequences from countries with suspected ZIKV infections^26^ can hinder accurate ZIKV identification and complicate surveillance attempts. For example, the generic qualifier “country” available for uploaded nucleotide sequences on GenBank is defined as the location pertaining to the isolation of the sequenced organism^27^, which may be interpreted as the location where sequencing of an organism took place as opposed to the location where an infection may have occurred. This is likely the case of Sample 3 – Caribbean in our sample set, which clusters within a group of samples with “country” specified as the USA. However, upon closer inspection, some of these ZIKV infections from the USA cluster were likely travel-related. For example, although the country for sample sequence KX842449 is recorded as USA, the sample is from an individual recently returning from Cuba. Additionally, the WHO reference sequence from French Polynesia (GenBank accession number: KX369547) (10,769 bp) used in many publications does not cover the full ZIKV genome (10,807 bp) and its protein length differs from the NCBI reference genome (GenBank accession number: NC_012532)^26^ derived from ZIKV strain MR 766. This may in turn complicate subsequent ZIKV protein annotations. Sequence databases may benefit from adopting additional features such as “suspected region of infection” for sequences of infectious pathogens, to maximize sequence utility and research reproducibility.

Additional analysis performed in this study also showed that > 30% of the GenBank reference sequences shared their closest relative with samples isolated from different countries. The substitution rate measured for ZIKV during the epidemic in the Americas is high compared to other flaviviruses^28^. Low genomic variability and sequence similarity observed in this study may be a result of the rapid early spread of ZIKV in the Americas in an immunologically naive population^16^, and the uneven sequencing of samples from endemic cases spatiotemporally^29^. In general, phylogenetic analysis of ZIKV may present unique challenges relative to other more rapidly-evolving viruses such as HCV.

Low genomic variability of ZIKV may present additional obstacles during global surveillance of ZIKV. Although the five Canadian ZIKV samples had five different sources of infections, there were high sequence similarities between the samples and the WHO reference sequence with respect to the identification of minority, non-consensus nucleotides. The diversity of ZIKV was also substantially lower relative to HCV suggesting limited, evolutionary selective pressures. Large segments of highly conserved regions of the genome, lack of an RNA dependent RNA polymerase with similar fidelity of RNA synthesis compared to HCV, and lack of drug pressure may be reasons for the low genomic variability observed^30,31^. Fortunately, low genomic variation is likely an advantage in vaccine and therapeutic developments^32^.

While investigating the genetic diversity of all ZIKV sequences based on country and the global region where each sample was identified and/or sequenced, a fewer number of unique nucleotides were identified and lower variation were observed in the countries located in the Western Hemisphere. Our results further corroborate and support previous findings and predictions that indicate the evolutionary progression of ongoing epidemics may decrease as time passes^28^. Sequences from USA and neighboring regions such as Haiti and Dominican Republic have high sequence similarity, compared to sequences from Asian regions such as South Korea, Thailand, or Cambodia. Our findings reinforce the notion of splitting of the Asian lineage into two distinct lineages, American and Asian, as suggested by Gubler *et al*.^33^.

There are several limitations within our study that may be addressed by future research. Firstly, the low number of samples from Canadian travelers within our dataset may impact the validity of our findings regarding genomic variability of ZIKV. With a larger sample size or a greater global sampling, we will likely be able to better interpret the use of phylogenetic analysis in identifying origins of travel-acquired ZIKV infections. Perhaps one of the biggest limitations is due to the absence of whole-genome reference sequences available from countries associated with travel-related ZIKV infection. Although we were unable to confirm the country of infection for two of five samples due to missing reference sequences, we were able to identify the broad geographical region of infection. Low genomic variability of ZIKV within this dataset can potentially distort phylogenetic inference.

Surveillance of ZIKV is also dependent on healthcare providers, and their vigilant and persistent inspection of individuals who may have been exposed to ZIKV. Even individuals who are asymptomatic can potentially transmit ZIKV to unsuspecting partners through sexual transmission^34,35^. Continual surveillance of ZIKV is essential to early detection of ZIKV outbreaks, particularly in areas where *Aedes* mosquito populations are extant and growing. While WGS of ZIKV may present obstacles during epidemic surveillance, mostly due to lack of standardized available data during phylogenetic analysis and low ZIKV genomic diversity, it provides a unique method of identifying broad geographical regions of an infection and can also provide insight into the genetic variability of a circulating virus.

## Methods

### Study population

Specimens from five subjects with confirmed travel-acquired ZIKV infection (putatively from Belize, Mexico, an undisclosed Caribbean region, Barbados, and Panama) were obtained from the British Columbia (BC) Centre for Disease Control (BCCDC) Public Health Laboratory and the samples had a range of cycle threshold (Ct) values (21–33).

Three longitudinal whole-genome HCV sequences were provided from one individual living with HCV genotype 1a strain from the BC Centre for Substance Use’s Vancouver Injection Drug Users Study (VIDUS), a prospective ongoing cohort established in 1996 involving people who inject drugs. The VIDUS participants provide clinical and demographic data through completion of questionnaires at bi-annual clinical visits which also involve HCV and human immunodeficiency virus (HIV) tests^36^.

The University of British Columbia Providence Health Care Research Ethics Board granted ethical approval for this study (H16-02865). All experiments were performed in accordance with institutional guidelines and regulations. A waiver of consent was obtained as research was conducted on anonymized leftover, stored clinical specimens and there were no direct benefits to the participants as infections had previously been diagnosed and reported.

### Whole-genome sequencing

Confirmation of ZIKV infection and viral nucleic acid extraction occurred at the BCCDC while RT-PCR, WGS, and data analysis occurred at the BC Centre for Excellence in HIV/AIDS (BC-CfE) in Vancouver, BC, Canada. Detailed description of the methodology and primer sequences used can be found elsewhere^37^. Briefly, sequencing of ZIKV genome was performed on an Illumina MiSeq platform (San Diego, California, United States) using a previously published procedure designed to overcome some of the limitations of low viral RNA copy number and partially degraded samples by amplifying several short amplicons to create a tiling path across the ZIKV genome^37^. This methodology involves a multiplex PCR system which amplifies alternating regions of the ZIKV genome using two separate primer pools. The purpose of this is to amplify approximately 400 nucleotide regions which overlap by 100 nucleotides, providing consistent coverage throughout the genome. In total, 35 primers in two separate primer pools were kindly provided by Dr. Nick Loman from the University of Birmingham to span the ZIKV genome (10,807 bp). KAPA Hyper Library preparation kit from Kapa Biosystems (Wilmington, Massachusetts, United States) and duel-indexed adapters from Illumina were used for MiSeq library construction^38^.

### Data analysis

#### Sequencing analysis

Sequences generated from the MiSeq were quality trimmed and primers were removed using Trimmomatic, aligned to each respective reference sequence using BWA-MEM, and processed with SAMtools^39,40^. Paired-end reads were aligned to a set of 17 phylogenetically distinct whole-genome reference sequences obtained from GenBank using the default settings for local alignment. This was followed by a re-alignment to the single reference with the highest overall read count for each sample and, subsequently using custom Python scripts, consensus sequences were obtained from MPILEUP files using base quality and mapping quality thresholds of 15. Custom Python scripts can be found at https://github.com/vmon5813/ZikaPhyloSurveillanceWGS. Sequencing data generated during this study are available in the GenBank repository. For HCV sequences, consensus sequences were similarly generated using an alternative reference database with full-length HCV genomes (n = 3,162) from the Los Alamos HCV database^41^.

#### Phylogenetic analysis

Phylogenetic analysis was performed to investigate geographic clustering of travel-acquired ZIKV cases. Whole-genome sequences were compiled into a reference set and were retrieved from GenBank in September 2018 (nucleotide search details: ‘zika[All Fields] AND “Zika virus”[porgn:__txid64320] AND (viruses[filter] AND (“9000”[SLEN]: “11000”[SLEN]))). In total, 864 nucleotide sequences ranging in length (9–11 kbp) were obtained in FASTA format. Nucleotide sequences with partial genomes, large repeated regions of ambiguous nucleotides, sequences with missing geographic origin, and confirmed non-ZIKV sequences were excluded from the dataset. In total, 362 sequences were used as reference sequences from different countries of interest (n = 39) (Supplementary Table S1). The United Nations geoscheme was used to identify geographical regions of the countries of interest^42^.

Canadian samples and GenBank reference sequences were aligned with the multiple sequence aligner MUSCLE^43^ using the parameters for large dataset alignment. Sequences were trimmed to WHO ZIKV reference sequence from French Polynesia (GenBank accession number: KX369547)^19^ for consistency, using alignment viewing program Aliview^44^. Phylogenetic trees were inferred in a maximum likelihood framework under a General Time Reversible (GTR) substitution model with gamma-distributed rate heterogeneity using RAxML version 8.2.12^45^. The reliability of the phylogenetic topology was estimated using a bootstrap analysis with 1000-pseudoreplicates. Tree topology and the relationship between taxa among different clades were observed. Clinical travel cases were predicted to be observed in the phylogeny most closely related to reference sequences from the same geographical regions.

#### Genomic diversity of travel-acquired ZIKV infections

Variation across the ZIKV genome was measured for each nucleotide position with a minimum read depth of 100. To compare the overall fraction of minority variants with the consensus nucleotide at each position relative to the WHO ZIKV reference sequence from French Polynesia (GenBank accession number: KX369547)^19^, the average count and percentage of all variants for each sample was calculated for each gene and at each position, respectively, and plotted in R using the ggplot2 package^46^. Variants present at >99% and >1% of the reads mapped at a nucleotide position were classified as the consensus nucleotide and minority variants, respectively. Variants present at <1% were classified as noise.

Furthermore, a Shannon diversity index was also used to characterize and compare the intrafamilial genomic diversity of ZIKV to the HCV sequences acquired from one individual sampled longitudinally at three timepoints. Shannon diversities were then calculated per position from the nucleotide proportions using custom Python scripts (Supplementary Equation (1)), which can be found at https://www.https://github.com/vmon5813/ZikaPhyloSurveillanceWGS. A sliding window approach was used to highlight diversification across all genomic motifs where diversities at each position were summed over each 100 bp window^47,48^. These were then plotted in R using the ggplot2 package^46^.

#### Country-specific genomic diversity of whole-genome ZIKV sequences

Patristic distances for the phylogenetic tree were obtained using the Python package DendroPy^49^. Countries with smaller intra-country patristic distances relative to inter-country patristic distances indicated country-specific sequences were more closely related to each other compared with other between-country sequences. The countries for each sequence and its closest relative were also identified and compared.

To assess genomic variation of current circulating ZIKV strains based on geographical region, variable nucleotides unique to each country were identified. A sequence alignment trimmed to protein-coding regions of ZIKV was performed on the genomes dated after 2000 from the reference dataset, as well as on the travel-related ZIKV samples. A consensus sequence was obtained for each country that had > 1 whole-genome sequence (n = 24) using Geneious Prime version 2019.2.1 (https://www.geneious.com) and countries with a single representative sequence were also included in the analysis (n = 12). Countries with reference sequences acquired prior to the year 2000 were excluded (n = 5). Variable positions were initially identified if they were present in >50% of the reference sequences corresponding to a specific country, while not identified in consensus sequences corresponding to any other country in the dataset. In order to determine all unique variants, each genomic position with a redundant base other than “N” was accounted for in this analysis by counting all nucleotides signified by each base. Subsequently, all of the positions were further screened to identify country-specific unique variants relative to any individual sequences from alternative countries by comparing each variant to all nucleotides at the respective position.

### Disclaimer

Preliminary data was presented at the American Society for Microbiology Conference on Rapid Applied Microbial Next-Generation Sequencing and Bioinformatic Pipelines in Washington, DC in September 2018, by K.K (abstract number: 120).

## Supplementary information

Supplemental.Phylogenetic surveillance of travel-related Zika virus infections through whole-genome sequencing methods
