# Supplementary material for: Phylogenetic surveillance of travel-related Zika virus infections through whole-genome sequencing methods
Source: Sci Rep. 2019 Nov 11;9:16433. doi: 10.1038/s41598-019-52613-8 (PMC6848190; doi:10.1038/s41598-019-52613-8)
Supplement: Supplementary file 1 — Supplemental.Phylogenetic surveillance of travel-related Zika virus infections through whole-genome sequencing methods [file 41598_2019_52613_MOESM1_ESM.pdf]

Title: Phylogenetic surveillance of travel-related Zika virus infections through whole-genome sequencing methods

Authors:

Kimia KAMELIAN [1, 2]

Vincent MONTOYA [2]

Andrea OLMSTEAD [2]

Winnie DONG [2]

Richard HARRIGAN [1]

Muhammad MORSHED [3, 4]

Jeffrey B JOY [1, 2] *

Affiliations:

1. University of British Columbia, Division of AIDS, Department of Medicine, Vancouver BC, Canada
2. BC Centre for Excellence in HIV/AIDS, Vancouver BC, Canada
3. BC Centre for Disease Control Public Health Laboratory, Vancouver BC, Canada
4. University of British Columbia, Department of Pathology and Laboratory Medicine, Vancouver BC, Canada

Running Head: Surveillance of Zika virus

*** Corresponding Author:**

Jeffrey B Joy

E-mail: jjoy@cfenet.ubc.ca, Telephone: 604-368-5569

University of British Columbia, Division of AIDS, Department of Medicine, Vancouver BC, Canada

Supplementary Fig. S1. Average proportion of nucleotide variants present in Canadian travel-acquired ZIKV samples.

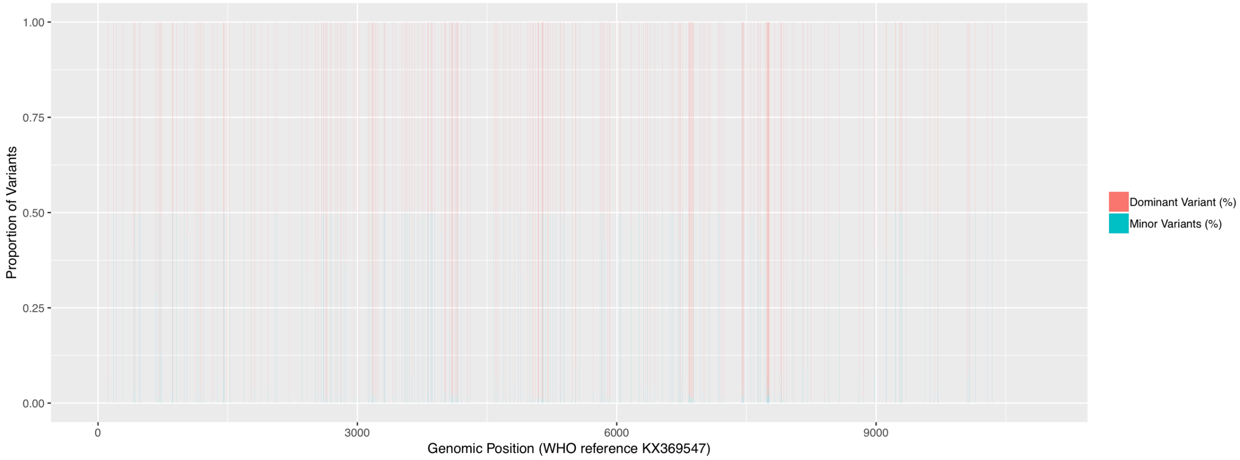

Supplementary Table S1. Whole-genome ZIKV reference set collected from GenBank.

Accession Number	Country	Date of Isolation
KU963574	NIGERIA	1968
HQ234500	NIGERIA	1968
HQ234501	SENEGAL	1984
KU955595	SENEGAL	1984
KU955592	SENEGAL	1984
KU955591	SENEGAL	1984
KX601166	SENEGAL	1984
KY348860	SENEGAL	1984
KF268948	CENTRAL-AFRICAN-REPUBLIC	1976
MH130104	UGANDA	1947
MH130097	UGANDA	1947
MH130105	UGANDA	1947
MH130102	UGANDA	1947
KU955594	UGANDA	1947
MH130100	UGANDA	1947
MH130094	UGANDA	1947
MH130109	UGANDA	1947
MH130101	UGANDA	1947
MH130095	UGANDA	1947
MH130106	UGANDA	1947
MH130103	UGANDA	1947
MH130098	UGANDA	1947
MH130096	UGANDA	1947
MH130108	UGANDA	1947
MH130099	UGANDA	1947
MH130107	UGANDA	1947
KX377336	MALAYSIA	1966
KX694533	MALAYSIA	1966
HQ234499	MALAYSIA	1966
KX601167	MALAYSIA	1966
KU681082	PHILIPPINES	2012
KY120353	SOUTH-KOREA	2016
KY553111	SOUTH-KOREA	2016
EU545988	MICRONESIA	2007
MG645981	THAILAND	2006

MH368551	CAMBODIA	2016
MH158236	CAMBODIA	2010
KU955593	CAMBODIA	2010
JN860885	CAMBODIA	2010
KY272987	THAILAND	2016
KF993678	CANADA	2013
KX694532	THAILAND	2013
KY328290	CHINA	2016
KX051562	THAILAND	2015
MH119185	THAILAND	2016
KX051561	THAILAND	2013
KU179098	INDONESIA	2014
KX051560	THAILAND	2013
MG807647	THAILAND	2017
KY126351	THAILAND	2016
MH013290	THAILAND	2017
LC369584	THAILAND	2017
MG548661	THAILAND	2016
MG548660	THAILAND	2016
MF996804	THAILAND	2017
MF692778	THAILAND	2016
MG807646	THAILAND	2016
KY241778	SINGAPORE	2016
KY241777	SINGAPORE	2016
KY241779	SINGAPORE	2016
KY241780	SINGAPORE	2016
KY241776	SINGAPORE	2016
MH255601	SINGAPORE	2016
KY241783	SINGAPORE	2016
KY241781	SINGAPORE	2016
KY241782	SINGAPORE	2016
KY241787	SINGAPORE	2016
KY241775	SINGAPORE	2016
KY241786	SINGAPORE	2016
KY241785	SINGAPORE	2016
KY241784	SINGAPORE	2016
KU681081	THAILAND	2014
LC219720	JAPAN	2016
MF801384	HONDURAS	2016
KU744693	VENEZUELA	2016

KY241788	SINGAPORE	2016
MF801414	MEXICO	2016
MF801417	MEXICO	2016
KX827268	USA	2016
MF098771	MEXICO	2017
MH157208	MEXICO	2016
MH157213	MEXICO	2016
KY631494	MEXICO	2015
KY631493	MEXICO	2015
MF801398	MEXICO	2016
MF801413	MEXICO	2016
KX856011	MEXICO	2016
MH157202	MEXICO	2016
KY120349	MEXICO	2016
MF801395	MEXICO	2016
KY120348	MEXICO	2016
KX247632	MEXICO	2015
MF434516	NICARAGUA	2016
MF801397	MEXICO	2016
MF801396	MEXICO	2016
KY785442	HONDURAS	2016
KY765324	NICARAGUA	2016
KY785448	HONDURAS	2016
MF801406	MEXICO	2016
MF434522	NICARAGUA	2016
MF801378	GUATEMALA	2016
MF801403	MEXICO	2016
KY014306	HONDURAS	2016
MF988734	SINGAPORE	2017
MH063262	CUBA	2017
MF159531	CUBA	2017
MF801418	MEXICO	2016
KY693676	HONDURAS	2016
MF434517	NICARAGUA	2016
KU870645	USA	2016
KY014319	HONDURAS	2016
KY765322	NICARAGUA	2016
KY765317	NICARAGUA	2016
KY765321	NICARAGUA	2016
KY765318	NICARAGUA	2016

MF434521	NICARAGUA	2016
KY014312	HONDURAS	2016
KY328289	HONDURAS	2016
MF801410	MEXICO	2016
MF593625	CHINA	2016
KY927808	CHINA	2016
MF801387	HONDURAS	2016
MF801402	MEXICO	2016
MF801412	MEXICO	2016
MF801426	NICARAGUA	2016
KU501217	GUATEMALA	2015
KU501216	GUATEMALA	2015
KY765326	NICARAGUA	2016
KY765323	NICARAGUA	2016
KY765320	NICARAGUA	2016
KX421195	NICARAGUA	2016
KX421194	NICARAGUA	2016
KX906952	HONDURAS	2016
KX694534	HONDURAS	2015
KX262887	HONDURAS	2016
KY765327	NICARAGUA	2016
KY765325	NICARAGUA	2016
KY693677	HONDURAS	2016
KY785418	HONDURAS	2016
KY014315	HONDURAS	2016
KY559021	BRAZIL	2016
MH063261	CUBA	2017
LC191864	JAPAN	2016
KY785435	DOMINICAN-REPUBLIC	2016
KY785415	DOMINICAN-REPUBLIC	2016
MF438286	CUBA	2017
KY785475	DOMINICAN-REPUBLIC	2016
MH063264	CUBA	2017
LC331561	JAPAN	2016
KY785422	USA	2016
KY785476	DOMINICAN-REPUBLIC	2016
KY014314	DOMINICAN-REPUBLIC	2016
MF098769	DOMINICAN-REPUBLIC	2016
MF098768	DOMINICAN-REPUBLIC	2016
MF664436	DOMINICAN-REPUBLIC	2016

KY014299	USA	2016
KX922708	USA	2016
KX922705	USA	2016
KY014322	USA	2016
KX838906	USA	2016
KY014302	DOMINICAN-REPUBLIC	2016
KY785441	DOMINICAN-REPUBLIC	2016
KY785468	USA	2016
KY014324	USA	2016
KX838904	USA	2016
KX832731	USA	2016
KY014325	USA	2016
KX922704	USA	2016
KX922706	USA	2016
KX922703	USA	2016
KY014323	USA	2016
KX838905	USA	2016
KY014295	USA	2016
KX842449	USA	2016
KY014316	USA	2016
KX922707	USA	2016
KX673530	UK	2016
MF384325	HAITI	2016
KY415991	HAITI	2014
KY415989	HAITI	2014
KY415988	HAITI	2014
KY415990	HAITI	2014
KY415987	HAITI	2014
KY415986	HAITI	2014
KY014321	DOMINICAN-REPUBLIC	2016
KY014305	DOMINICAN-REPUBLIC	2016
KY014318	DOMINICAN-REPUBLIC	2016
KY014300	DOMINICAN-REPUBLIC	2016
MF098765	DOMINICAN-REPUBLIC	2016
MF098766	DOMINICAN-REPUBLIC	2016
LC190723	JAPAN	2016
MF098764	DOMINICAN-REPUBLIC	2016
KY785420	DOMINICAN-REPUBLIC	2016
KY014304	DOMINICAN-REPUBLIC	2016
KX269878	ITALY	2016

KU853013	ITALY	2016
KU853012	ITALY	2016
KX520666	BRAZIL	2015
KY003154	ITALY	2016
KY003153	ITALY	2016
KY441401	BRAZIL	2016
MF098767	RUSSIA	2016
KY693680	VENEZUELA	2016
KY558996	BRAZIL	2015
KU926310	BRAZIL	2016
KY785455	BRAZIL	2016
KY559013	BRAZIL	2016
KY559007	BRAZIL	2016
KY559005	BRAZIL	2016
KY120352	BRAZIL	2016
KU729217	BRAZIL	2015
MF073359	BRAZIL	2015
MF073358	BRAZIL	2015
KY631492	BRAZIL	2016
KX056898	CHINA	2016
KY379148	CHINA	2016
MF167360	CHINA	2016
KU955590	CHINA	2016
KX766028	DOMINICAN-REPUBLIC	2016
KU740184	CHINA	2016
KU761564	CHINA	2016
KU820898	CHINA	2016
KY559027	BRAZIL	2016
KU497555	BRAZIL	2015
KY693679	PERU	2016
KY693678	PERU	2016
KY785466	COLOMBIA	2016
KX548902	COLOMBIA	2015
MH544701	COLOMBIA	2016
MF574561	COLOMBIA	2015
MF574555	COLOMBIA	2015
KX893855	VENEZUELA	2016
KX702400	VENEZUELA	2016
MF574575	COLOMBIA	2015
MF574567	COLOMBIA	2015

MF574576	COLOMBIA	2015
MF574560	COLOMBIA	2015
MF574568	COLOMBIA	2015
MF574572	COLOMBIA	2015
MF574559	COLOMBIA	2015
MF574562	COLOMBIA	2015
MF574556	COLOMBIA	2015
MF574558	COLOMBIA	2015
MF574566	COLOMBIA	2015
MF574565	COLOMBIA	2015
MF574571	COLOMBIA	2015
MF574573	COLOMBIA	2015
MF574569	COLOMBIA	2015
MF574563	COLOMBIA	2015
MF574564	COLOMBIA	2015
MF574570	COLOMBIA	2015
MF574557	COLOMBIA	2015
KX247646	COLOMBIA	2016
MF574554	COLOMBIA	2015
KX087102	COLOMBIA	2015
KU820897	COLOMBIA	2015
MF574553	COLOMBIA	2015
MF574583	COLOMBIA	2015
MF574588	COLOMBIA	2015
MF574586	COLOMBIA	2015
MF574584	COLOMBIA	2015
MF574582	COLOMBIA	2015
MF574581	COLOMBIA	2015
MF574580	COLOMBIA	2015
MF574577	COLOMBIA	2015
MF574574	COLOMBIA	2015
MF574552	COLOMBIA	2015
KY317937	COLOMBIA	2016
KU647676	MARTINIQUE	2015
KU922960	MEXICO	2016
KU922923	MEXICO	2016
KY785469	COLOMBIA	2016
KY989971	COLOMBIA	2015
KX156775	PANAMA	2015
KX156776	PANAMA	2015

MF574585	COLOMBIA	2015
MF574587	COLOMBIA	2015
KX156774	PANAMA	2015
KY317940	COLOMBIA	2016
KY317939	COLOMBIA	2016
KY317936	COLOMBIA	2016
KY014303	DOMINICAN-REPUBLIC	2016
KY317938	COLOMBIA	2016
KX198135	PANAMA	2016
NC035889	BRAZIL	2015
KU527068	BRAZIL	2015
MF099651	CHINA	2016
MF964216	CHINA	2016
KU761560	CHINA	2016
KU761561	CHINA	2016
MF036115	CHINA	2016
KY967711	CHINA	2016
MH055376	CHINA	2016
MG674719	CHINA	2016
MG674718	CHINA	2016
KX266255	CHINA	2016
KX253996	CHINA	2016
KU955589	CHINA	2016
KU820899	CHINA	2016
KX185891	CHINA	2016
KU963796	CHINA	2016
KU866423	CHINA	2016
KU997667	CHINA	2016
KX013000	CHINA	2016
KX117076	CHINA	2016
KY014320	BRAZIL	2016
KY014296	BRAZIL	2016
KX879603	ECUADOR	2016
KX879604	ECUADOR	2016
MF794971	ECUADOR	2016
KU926309	BRAZIL	2016
KY272991	BRAZIL	2016
KU312312	SURINAME	2015
KY785464	PUERTO-RICO	2016
MH158237	PUERTO-RICO	2015

KX377337	PUERTO-RICO	2015
MF574578	COLOMBIA	2015
KU501215	PUERTO-RICO	2015
KX601168	PUERTO-RICO	2015
MF574579	COLOMBIA	2015
KX087101	PUERTO-RICO	2015
KY348640	SURINAME	2016
KY785450	BRAZIL	2016
KY014297	BRAZIL	2016
MF073357	BRAZIL	2016
KY441403	BRAZIL	2016
KY441402	BRAZIL	2016
KU365778	BRAZIL	2015
KU758877	FRENCH-GUIANA	2015
KU937936	SURINAME	2016
KY014317	BRAZIL	2016
KY559015	BRAZIL	2016
KX806557	AUSTRALIA	2016
KU707826	BRAZIL	2015
KU365779	BRAZIL	2015
KU365780	BRAZIL	2015
KU365777	BRAZIL	2015
KX280026	BRAZIL	2015
KX811222	BRAZIL	2016
KU729218	BRAZIL	2015
KX830930	BRAZIL	2016
KX197205	BRAZIL	2015
KU991811	ITALY	2016
KX447517	FRENCH-POLYNESIA	2014
KX051563	USA	2016
KU509998	HAITI	2014
KU321639	BRAZIL	2015
KX197192	BRAZIL	2015
KR872956	BRAZIL	2016
MF352141	BRAZIL	2015
KY558999	BRAZIL	2016
KX447510	FRENCH-POLYNESIA	2013
KX447516	FRENCH-POLYNESIA	2014
KX447511	FRENCH-POLYNESIA	2014
KX447515	FRENCH-POLYNESIA	2013

KX447513	FRENCH-POLYNESIA	2013
KX447512	FRENCH-POLYNESIA	2013
MG827392	FRENCH-POLYNESIA	2013
KX369547	FRENCH-POLYNESIA	2013
KX447514	FRENCH-POLYNESIA	2014
KJ776791	FRENCH-POLYNESIA	2013
KX447509	FRENCH-POLYNESIA	2013

Supplementary Equation (1). Equation used for Shannon diversity calculation.

The proportion (p_i) of each nucleotide at each position is multiplied by the natural logarithm of the same respective proportion and summed over all four nucleotides.

$$SE = - \sum_{i=0}^{4} p_i \, ln \, p_i$$
